# Temperature evolution of dense gold and diamond heated by energetic laser-driven aluminum ions

**DOI:** 10.1038/s41598-022-18758-9

**Published:** 2022-09-07

**Authors:** C. Song, S. Lee, W. Bang

**Affiliations:** 1 Department of Physics and Photon Science, GIST, Gwangju, 61005 South Korea; 2 Center for Relativistic Laser Science, Institute for Basic Science, Gwangju, 61005 South Korea

**Keywords:** Physics, Plasma physics, Laser-produced plasmas

## Abstract

Recent studies have shown that energetic laser-driven ions with some energy spread can heat small solid-density samples uniformly. The balance among the energy losses of the ions with different kinetic energies results in uniform heating. Although heating with an energetic laser-driven ion beam is completed within a nanosecond and is often considered sufficiently fast, it is not instantaneous. Here we present a theoretical study of the temporal evolution of the temperature of solid-density gold and diamond samples heated by a quasimonoenergetic aluminum ion beam. We calculate the temporal evolution of the predicted temperatures of the samples using the available stopping power data and the SESAME equation-of-state tables. We find that the temperature distribution is initially very uniform, which becomes less uniform during the heating process. Then, the temperature uniformity gradually improves, and a good temperature uniformity is obtained toward the end of the heating process.

## Introduction

The acceleration of ions using modern high-power laser systems has led to the development of intense ion sources with high kinetic energy^1–6^. Laser-driven ions with speeds up to a few tens of percent of the light speed have been generated experimentally, carrying several tens of MeV/nucleon^6–11^. For example, laser-driven protons with maximum kinetic energy approaching 100 MeV have been demonstrated in recent experiments^9–11^. These laser-driven protons or ions transfer their kinetic energy to a sample very rapidly via Coulomb collisions before significant hydrodynamic expansion of the sample occurs^12–15^. The heated sample often reaches high temperatures above 10,000 K^16–20^, while still maintaining near-solid density. Because of these properties, laser-driven ions can be used in research areas such as the study of warm dense matter^17–20^ and fast ignition^21,22^.

Since temperature gradients within a sample make it difficult to analyze the measured physical properties of a heated sample, it is desirable to heat the sample uniformly to study its physical properties^16^. However, typical laser-driven ions heat the front surface of the sample preferentially because they exhibit a Maxwellian energy distribution^6,23^, in which less energetic ions predominate. Low-energy ions transfer all their kinetic energy and stop near the front surface of the sample. In contrast, more energetic ions mainly deposit their kinetic energy around the rear surface of the sample. They transfer only a small fraction of their kinetic energy before reaching their Bragg peaks^24^, where most of the energy transfer occurs. The energy transferred around the front surface of the sample is greater than the energy transferred near the rear surface because the number of less energetic ions is larger than the number of more energetic ions for Maxwellian energy distribution.

Laser-driven ions with some energy spread have been studied experimentally^1–3,25,26^ and theoretically^15,27,28^. For an ion beam with some energy spread, uniform heating can be achieved as a result of the balance between the energy transferred from the low-energy ions and the energy transferred from the high-energy ions. Recent studies^12,16^ have shown that a high energy laser-driven aluminum ion beam^3,29^ with some energy spread can heat small solid-density samples fairly uniformly to temperatures above 10,000 K.

While previous studies suggest good temperature uniformity of the resulting warm dense matter samples^12,16^, no study has examined temperature uniformity during heating. It is quite possible that the temperature uniformity is poor at the beginning or in the middle of the heating process. For example, the temperature uniformity of the sample might be poor in the middle of the heating process because high-energy ions transfer more kinetic energy to the rear surface of the sample than to the front. The temperature distribution becomes more uniform when low-energy ions reach the sample and heat the front surface.

Here we study the temporal evolution of the calculated temperatures of dense gold and diamond samples heated by a laser-driven aluminum ion beam in Ref.^12^. We use the Monte Carlo simulation code, SRIM^30^, and SESAME equation-of-state (EOS) tables^31–35^ to calculate the expected temperatures of the samples at different times. Based on these calculations, we investigate the temperature uniformity of the heated solid-density samples during the entire heating process.

## Simulation methods

**Figure 1:**
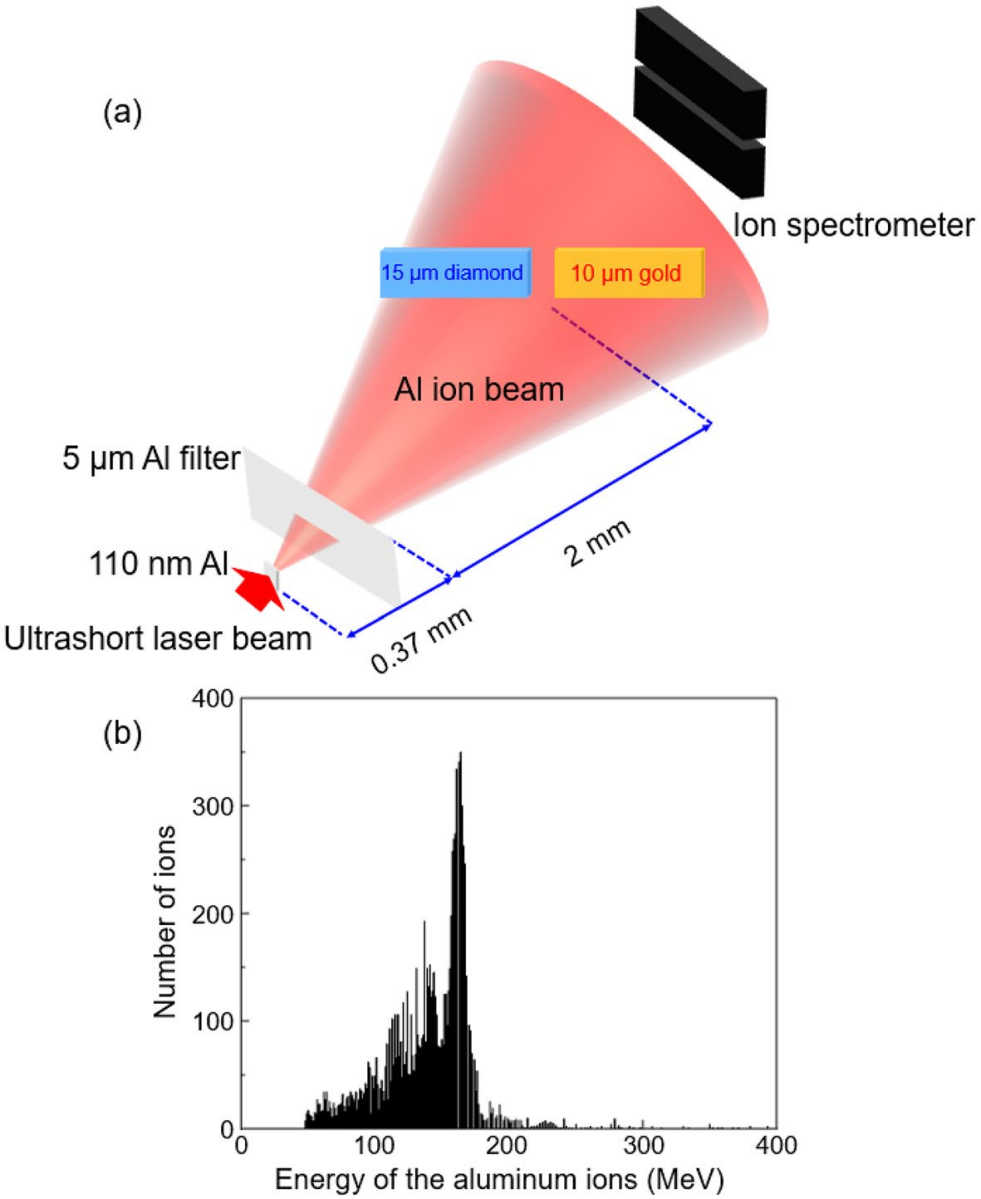
(**a**) A laser-driven aluminum ion beam with some energy spread impinges upon gold and diamond samples at a 45° incidence angle. The laser-driven aluminum beam heats gold and diamond samples isochorically. (**b**) Energy spectrum of the incident aluminum ions measured from Ref.^3^, which is used in our SRIM calculations.

Figure 1a shows a laser-driven aluminum ion beam^3^ incident on gold and diamond samples^12,16^. After an intense (~ 2 × 10^20^ W/cm^2^) laser pulse irradiates a 110-nm-thick aluminum foil, an energetic aluminum ion beam with some energy spread is generated^3^. The laser-driven aluminum ion beam diverged with a 20° cone half angle^3^, and impinged upon 10-μm-thick gold and 15-μm-thick diamond samples after traveling a source-to-sample distance of 2.37 mm at an incidence angle of 45°^12,16^. A 5 μm thick aluminum filter, inserted 0.37 mm behind the source and 2.0 mm before the samples, blocked any laser light passing through the 110 nm Al foil as well as low-energy protons (< 0.5 MeV) and low-energy aluminum ions (< 10 MeV)^12,16^.

In Fig. 1b, the black bars indicate the input data to our SRIM simulations, which represent the energy spectrum of 10,000 aluminum ions incident on gold and diamond samples. The average kinetic energy of the aluminum ions is 140 (± 33) MeV in Fig. 1b, and the input energy spectrum is based on a typical energy spectrum measured in Ref.^3^ using a Thompson parabola ion spectrometer.

## Results and discussion

We can calculate the energy deposited on the samples at different times by using the stopping power data from SRIM and the measured energy spectrum of the incident aluminum ions shown in Fig. 1b. Note that the arrival time of a single aluminum ion decreases with increasing kinetic energy. For example, a 200 MeV aluminum ion reaches gold or diamond 63 ps after the laser pulse irradiates the aluminum foil, whereas it takes 125 ps for a 50 MeV aluminum ion to travel the same distance.

**Figure 2:**
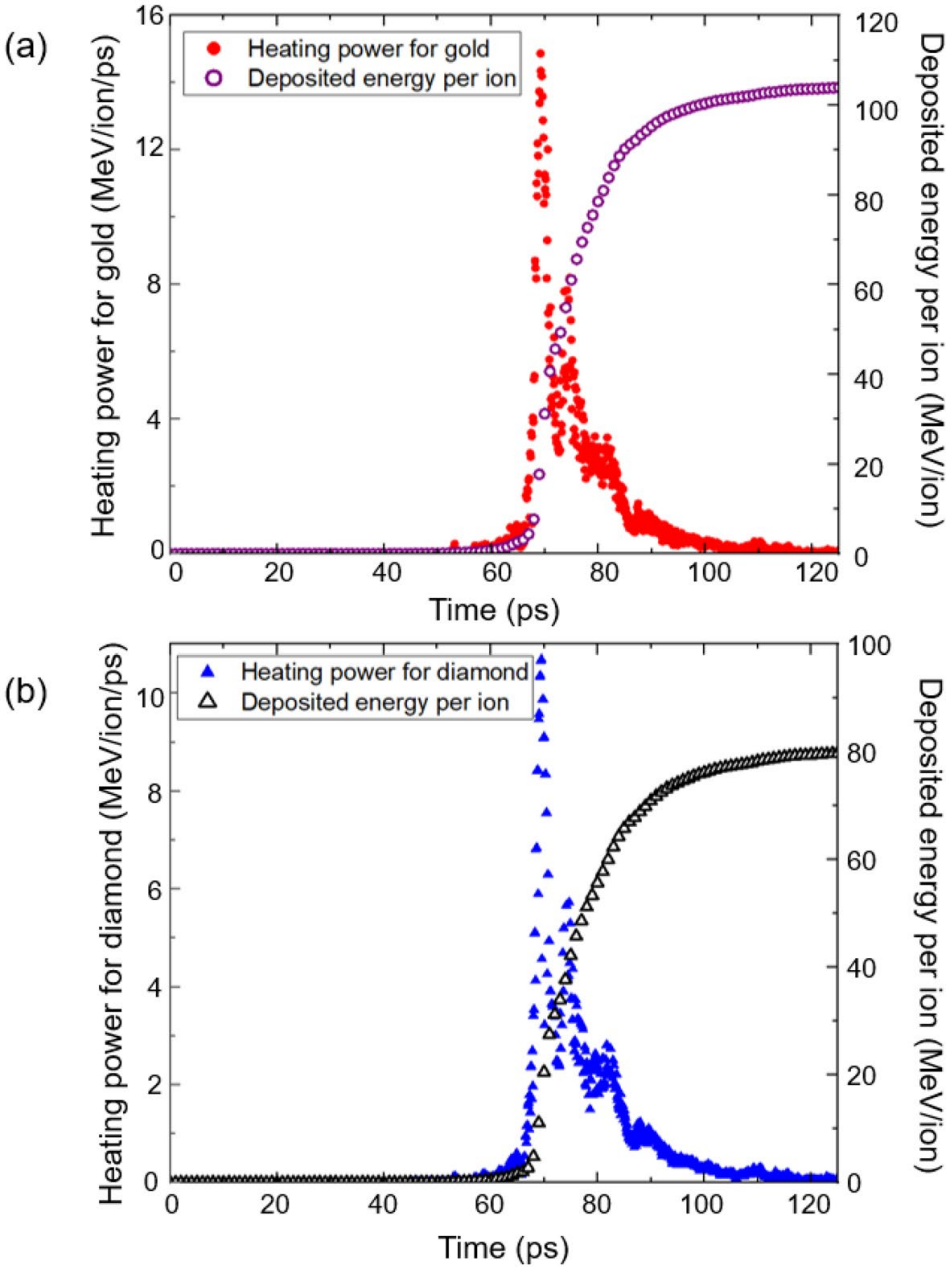
(**a**) The heating power of the incident aluminum ions for a 10-μm-thick gold sample at an angle of 45° is plotted as a function of time from 0 to 125 ps. The deposited energy per ion is also shown as a function of time. (**b**) The heating power for a 15-μm-thick diamond sample at an angle of 45° is shown as a function of time from 0–125 ps. The deposited energy per ion is also shown as a function of time.

Figure 2a and b show the heating powers and the deposited energy per ion for a 10-μm-thick gold sample and a 15-μm-thick diamond sample as a function of time. The solid red circles show the heating power for gold and the hollow purple circles indicate the deposited energy per ion in Fig. 2a. In Fig. 2b, the solid blue triangles show the heating power for diamond and the hollow black triangles indicate the deposited energy per ion. We define the heating power of the incident aluminum ions as the average deposited energy per ion per unit time. In Fig. 2a and b, we have accounted for the 45° incidence angle when calculating the corresponding travel times of the incident ions within the samples^12,16^.

The heating power remains negligible before 63 ps or until 200 MeV aluminum ions reach the samples. This is consistent with the tiny fraction (1.6%) of the aluminum ions above 200 MeV, as shown in Fig. 1b. Moreover, aluminum ions with this high kinetic energy lose only a small fraction of their kinetic energy because their ranges are larger than the sample thicknesses. For example, a 400 MeV aluminum ion transfers only 16% of its initial kinetic energy to the gold sample, whereas a 200 MeV aluminum ion transfers 47% of its kinetic energy.

In Fig. 2a, the heating power peaks at 69.2 ps, and most of the heating occurs during 68–86 ps. This time interval corresponds to aluminum ions with kinetic energy in the 107–173 MeV range, accounting for about 79% of the total incident ions shown in Fig. 1b. For gold, 107 MeV aluminum ions transfer all their kinetic energy to the sample, whereas 173 MeV aluminum ions transfer 101 MeV to the sample. After 86 ps, aluminum ions with kinetic energies less than 107 MeV reach the sample. These ions account for 16% of the total ions and stop within the sample after they have transferred all their kinetic energy. During the 86–125 ps interval, the heating power decreases to less than 1 MeV/ps for gold.

Figure 2b shows the heating power for the diamond sample, which is similar to the gold sample shown in Fig. 2a. The heating power for the diamond sample is slightly lower than that for the gold sample before 86 ps. For example, the heating power for the gold sample has a maximum of 14.9 MeV/ion/ps at 69.2 ps, while the heating power for the diamond sample has a maximum of 10.5 MeV/ion/ps at 69.5 ps. This is consistent with the larger deposited energy per ion for gold (= 104 MeV/ion at 125 ps) compared with that for diamond (= 80 MeV/ion at 125 ps). After 86 ps, however, the heating powers are almost equal because the aluminum ions transfer all their kinetic energy to both the gold and diamond samples.

**Figure 3:**
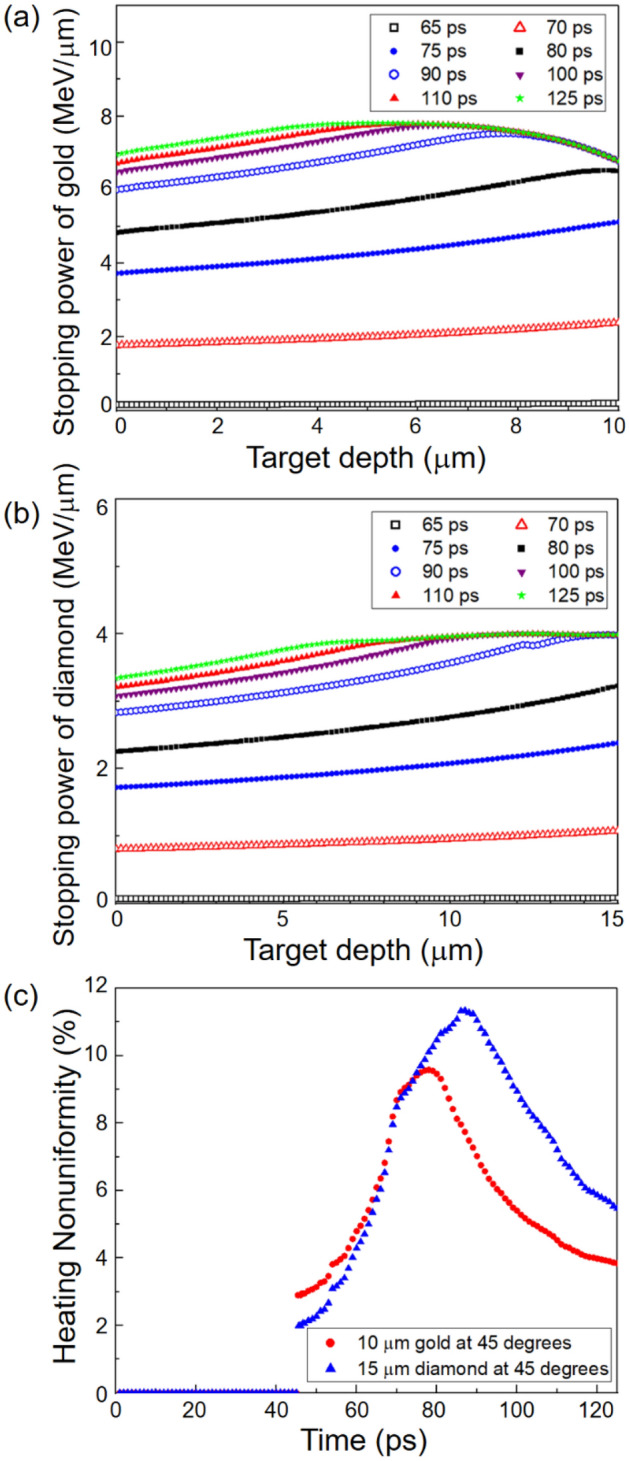
The time evolutions of (**a**) the stopping power of a 10-μm-thick gold sample at an angle of 45° and (**b**) the stopping power of a 15-μm-thick diamond sample at an angle of 45° are plotted as functions of the target depth from 65 to 125 ps. (**c**) The heating nonuniformities of the gold and diamond samples are plotted as functions of time in the 0–125 ps interval.

Figure 3a shows the time evolution of the stopping power of the 10 μm thick gold sample at an incidence angle of 45° from 65 ps (hollow black squares) to 125 ps (solid green stars). The stopping power of the gold sample mainly increases in the 65–90 ps (hollow blue circles) interval, which corresponds to the time interval where most of the heating occurs, as shown in Fig. 2a. For example, the stopping power of the gold sample at 90 ps is 6.87 (± 0.48) MeV/μm, which is 91% of the stopping power at 125 ps of 7.50 (± 0.29) MeV/μm.

Figure 3b shows the temporal evolution of the stopping power of the 15-μm-thick diamond sample at 45° at intervals of 65–125 ps. The stopping power of the diamond mainly increases in the interval of 65–90 ps, similar to the gold sample. However, the stopping power of the diamond sample is lower than that of the gold sample. For example, the stopping power of the diamond sample at 125 ps is 3.81 (± 0.21) MeV/μm.

In Fig. 3a and b, it can be seen how the uniformity of heating changes with time. To evaluate the degree of heating uniformity quantitatively, we follow the definition of heating nonuniformity in Ref.^16^

1 $${\text{Heating nonuniformity}} = \frac{{\text{Standard deviation of the stopping power}}}{{\text{Average stopping power}}} \times 100{ }\left( \text{\%} \right).$$

Figure 3c shows the heating nonuniformities from 0 to 125 ps for gold (solid red circles) and diamond (solid blue triangles), respectively. Initially, the heating appears to be fairly uniform for both the gold and diamond samples. For the gold sample, the heating nonuniformity worsens in the 45–78 ps interval. During this time interval, aluminum ions with kinetic energies greater than 130 MeV are incident on the gold sample. These energetic ions have ranges longer than the sample thickness and heat the rear surface more strongly as they slow down. Interestingly, the heating nonuniformity gradually improves in the time interval of 78–125 ps, which is due to more heating of the front and middle regions by less energetic ions. In the diamond sample, the heating nonuniformity increases until it reaches a maximum of 11.3% at 87 ps. Then, the heating nonuniformity gradually improves in the 87–125 ps interval, and becomes 5.6% at the end of the heating process.

For both the gold and diamond samples, the balance between the front surface heating by slower ions and rear surface heating by faster ions results in very uniform heating toward the end of the heating process, as shown in Fig. 3c. Throughout the heating process, the samples remain at solid density because the volume increase during heating is expected to be small (< 3%) based on the observed expansion speeds of the heated samples in Ref.^12^.

**Figure 4:**
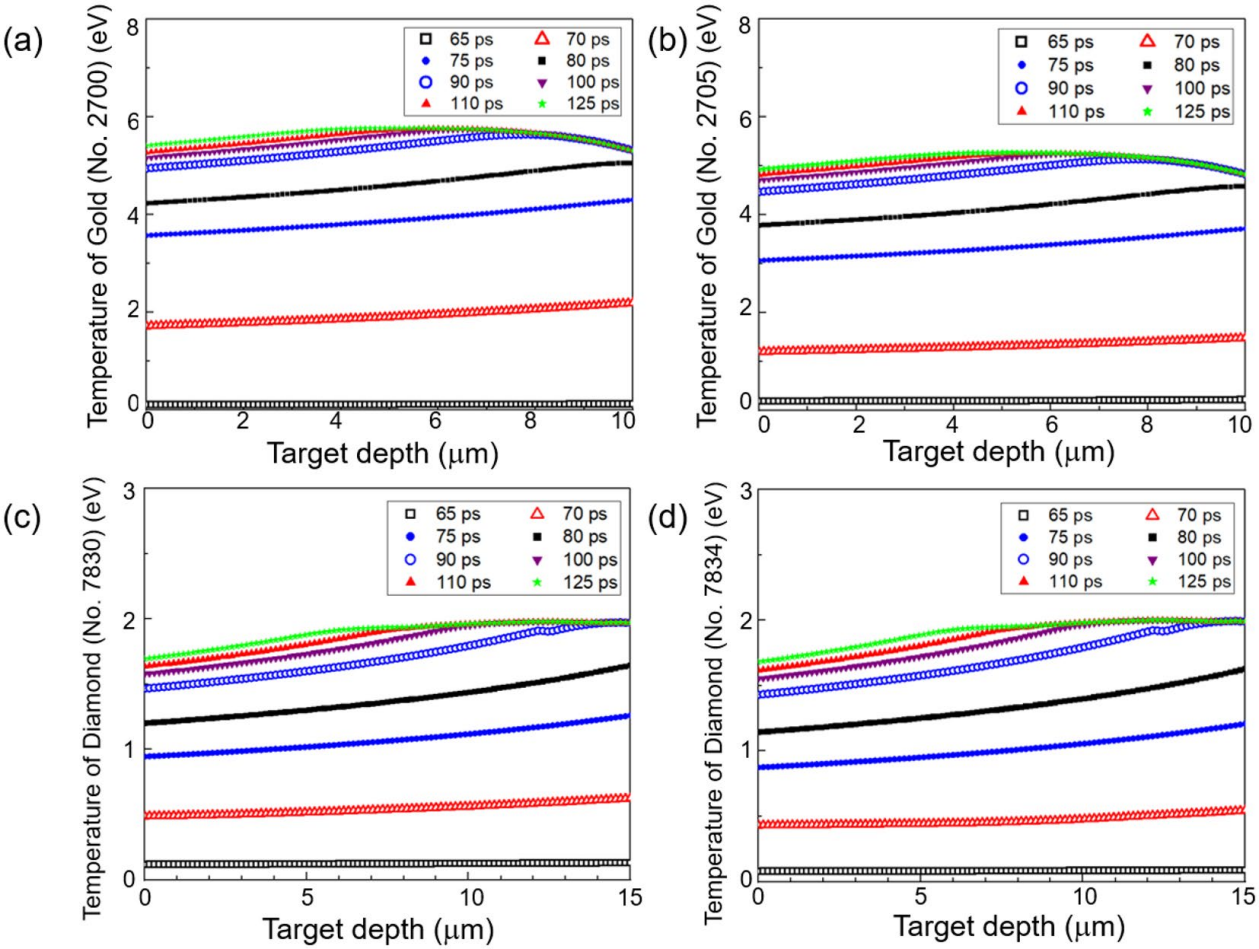
The temperature distribution within the 10-μm-thick gold sample is shown at different times from 65 to 125 ps. The temperatures are calculated using the SESAME EOS Tables (**a**) No. 2700 and (**b**) No. 2705. Similarly, the temperature distribution within the 15-μm-thick diamond is shown at different times using the SESAME EOS Tables (**c**) No. 7830 and (d) No. 7834.

Figure 4a–d show the temperature distribution within the 10-μm-thick gold sample and 15-μm-thick diamond sample at different times, from 65 to 125 ps. An incidence angle of 45° is considered in these calculations. We have calculated the temperature distribution of the heated gold and diamond samples using the cold stopping power data from SRIM and the corresponding SESAME EOS tables. We estimate that there can be up to 4% errors in our temperature calculations for gold and up to 2% errors for diamond because stopping powers are known to become larger for warm dense plasmas^36^. In Ref.^36^, a Bethe-style stopping power formula is presented for warm dense plasmas. Assuming a 10% decrease of the mean excitation energy for our warm dense gold and diamond samples, we estimate a stopping power increase of up to 4% for gold and up to 2% for diamond for a 100 MeV aluminum ion beam. Because the correction effect is insignificant and the mean excitation energy is unavailable for warm dense gold and diamond, we have used the cold stopping power data from SRIM in our calculations.

In Fig. 4a, SESAME EOS Table No. 2700 is used to calculate the temperature of the gold sample, whereas Table No. 2705 is used for the gold sample in Fig. 4b. Depending on the EOS tables used, the expected temperatures of the gold samples differ by about 10%, as shown in Fig. 4a and b. Specifically, the expected temperature of a 10-μm-thick gold sample using Table No. 2700 is 5.64 (± 0.13) eV at 125 ps, while the expected temperature using Table No. 2705 is 5.13 (± 0.12) eV. Note that the more recent SESAME Table No. 2705 ^33^ is known to predict the principal Hugoniot, thermal expansion, room-temperature isotherm, melt line, vapor pressure, and heat capacity of pure gold, which are substantially different from and superior to the corresponding predictions using Table No. 2700^16^.

Figure 4c and d show similar calculations for the 15-μm-thick diamond sample using SESAME EOS Tables No. 7830 and No. 7834, respectively. For the diamond sample, SESAME Tables No. 7830 and No. 7834 predict similar temperatures throughout the heating process. The expected temperature of the 15-μm-thick diamond sample at 125 ps using SESAME EOS Table No. 7830 is 1.89 (± 0.09) eV, while the predicted temperature using SESAME EOS Table No. 7834 is 1.91 (± 0.10) eV. The differences in the calculated temperatures using the two different EOS tables for the diamond sample are quite small throughout the heating process. Note that the radiation losses are insignificant in these calculations based on our estimates of the bremsstrahlung energy loss. This is because the bremsstrahlung optical depth of solid-density samples have relatively small values (< 0.1 μm) at temperatures on the order of several eV^37^. We estimate that the bremsstrahlung energy loss during 20 ps heating is less than 0.17% for the diamond sample at 1.9 eV.

To quantify the uniformity of the temperature distribution within the heated sample at different times, we define the temperature nonuniformity as^15^

2 $${\text{Temperature nonuniformity}} = \frac{{\text{Standard deviation of the temperature}}}{{\text{Average temperature}}} \times 100{ }\left( \text{\%} \right).$$

**Figure 5:**
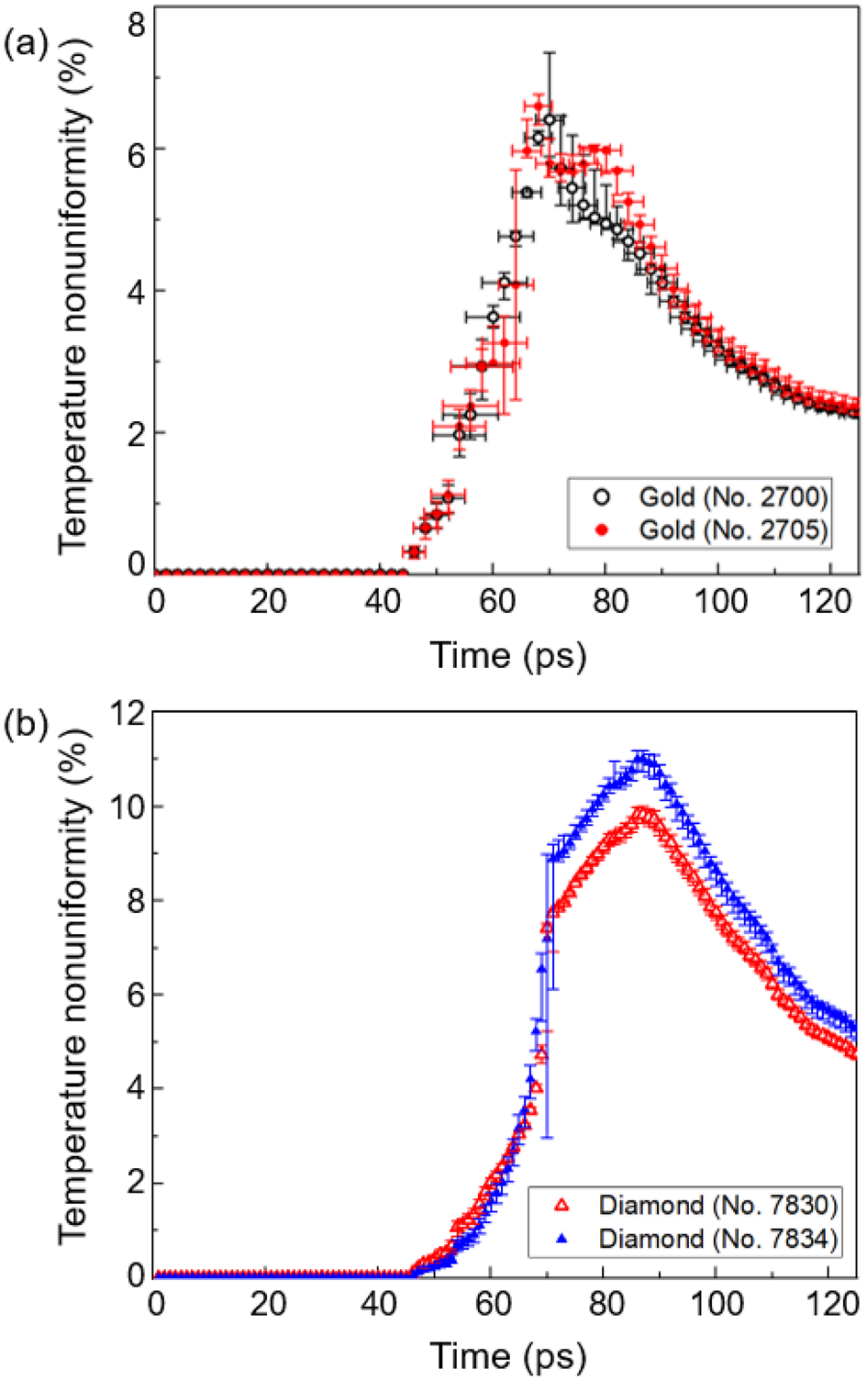
(**a**) Temperature nonuniformity of the gold sample is shown as a function of time from 0 to 125 ps. (**b**) Temperature nonuniformity of the diamond sample is shown as a function of time from 0 to 125 ps.

Figure 5a shows the time evolution of the temperature nonuniformity of the gold sample using SESAME EOS Tables No. 2700 (hollow black circles) and No. 2705 (solid red circles). The temperature nonuniformity of the gold sample calculated using SESAME EOS Tables No. 2700 and No. 2705 appears to be nearly identical. In both cases, the temperature uniformity deteriorates in the time interval of 45–80 ps. However, after 80 ps, the temperature uniformity gradually improves, reaching a temperature nonuniformity of 2–3% toward the end of the heating process.

Similarly, Fig. 5b shows the time evolution of the temperature nonuniformity of the diamond sample using SESAME EOS Tables No. 7830 (hollow red triangles) and No. 7834 (solid blue triangles). The temperature nonuniformity of the diamond sample increases up to 10–11% in the 45–87 ps interval, gradually improves after 87 ps, and becomes ~ 5% at the end of heating. The two EOS tables for diamond predict slightly different values, as shown in Fig. 5b.

The vertical error bars in Fig. 5a and b indicate the uncertainties in the expected temperatures of the gold and diamond samples, respectively, based on the reported ± 30% shot-to-shot fluctuation in the incident aluminum ion fluence^12,16^. In Fig. 5a, the horizontal error bars represent the estimated relaxation time for the gold sample, using the known electron–ion coupling factor for warm dense gold^38–41^. Based on these estimates, we expect local thermal equilibrium to be reached within several picoseconds, so the calculated temperatures in our figures represent both the electron and ion temperatures. In contrast, global thermal equilibrium is expected to be reached only after ~ 1 µs from heating for gold and ~ 20 µs for diamond based on our calculations of the diffusion coefficients of 5.6 eV gold and 1.9 eV diamond^12,16^. In other words, global thermal equilibrium is not reached within both gold and diamond samples on a nanosecond time scale relevant to this type of experiments. This explains why it is important to know the temperature distribution within the heated sample at different times during the heating process.

## Conclusion

We have studied the temporal evolution of the temperature distribution in gold and diamond samples heated with energetic quasimonoenergetic aluminum ion beams. While there have been previous studies suggesting good temperature uniformity of heated warm dense matter samples, no study has examined temperature uniformity during heating.

We have calculated the expected temperatures of the heated samples using SESAME EOS tables and the stopping power data from SRIM at different times. According to our simulation results, the temperature distribution within the heated solid-density sample is very uniform at the beginning, but becomes less uniform (7–11% nonuniformity) during the heating process for both the gold and diamond samples. Subsequently, the temperature uniformity gradually improves and a good temperature uniformity (2–5% nonuniformity) is achieved toward the end of the heating process. This study shows for the first time the evolution of the expected temperature distribution within warm dense gold and diamond samples during the heating process.

## Supplementary Information

Supplementary Information.

## Data Availability

The datasets used and/or analyzed during the current study are available from the corresponding author upon reasonable request.
