# Supplementary material for: Temperature evolution of dense gold and diamond heated by energetic laser-driven aluminum ions
Source: Sci Rep. 2022 Sep 7;12:15173. doi: 10.1038/s41598-022-18758-9 (PMC9452511; doi:10.1038/s41598-022-18758-9)
Supplement: Supplementary file 1 — Supplementary Information. [file 41598_2022_18758_MOESM1_ESM.docx]

Supplementary Information:

**Temperature evolution of dense gold and diamond heated by energetic laser-driven aluminum ions**

**C. Song^1, 2^, S. Lee^1, 2^, and W. Bang^1, 2^**

*^1^Department of Physics and Photon Science, GIST, Gwangju 61005, South Korea*

*^2^Center for Relativistic Laser Science, Institute for Basic Science, Gwangju 61005, South Korea*

We estimate that there can be up to 4% errors in our temperature calculations for gold and up to 2% errors for diamond as a result of stopping power increases in warm dense plasmas. In this section, we attempt to estimate the stopping power increase in the heated gold and diamond samples. Specifically, we estimate the stopping power errors using the Bethe-style stopping power formula presented in Ref. [36]. The Bethe-style stopping power formula can be written as [36]

$\frac{\mathrm{dE}}{\mathrm{dx}}=-\frac{4\pi Z_{t}^{2}}{m_{e}v_{t}^{2}}n_{e}\ln\left( \frac{2{m_{e}v}_{t}^{2}}{\overline{I}} \right),$ (1)

where *v_t_* is the velocity of the incident ion, *Z_t_* is the charge state of the incident ion, *m_e_* is the mass of an electron, *n_e_* is the electron number density of the sample, and $\overline{I}$ is the mean excitation energy of the sample material. For a 100 MeV aluminum ion, the maximum energy transfer term, *2m_e_v_t_^2^* is about 8.13 keV.

According to the NIST database, the mean excitation energy of gold is 790 eV, and the logarithm term in Eq. (1) becomes 2.33 for a 100 MeV aluminum ion. Even when we assume a 10% decrease of the mean excitation energy for our warm dense gold sample, we find the stopping power increases only by 4.5%.

Likewise, we can estimate the stopping power error for diamond. The mean excitation energy of carbon is 81 eV according to the NIST database. For a 100 MeV aluminum ion, the logarithm term for diamond in Eq. (1) becomes 4.61. Even if we assume a 10% decrease of the mean excitation energy for our warm dense diamond sample, we find the logarithm term becoming 4.71, which results in a 2.2% larger stopping power than the cold stopping power. Given that we have considered the worst case scenarios by assuming a 10% decrease of the mean excitation energy for our warm dense samples, we expect less than 4% errors in our temperature calculations for gold and less than 2% errors for diamond. These error estimates justify our use of the cold stopping power data from SRIM to calculate the temperature distributions within the heated gold and diamond samples.
